# Evaluation of Photoprotective Strategies in Asexual *Michelia guangdongensis* Lines Under High Temperature and Strong Light Stress Using the Entropy-Weighted TOPSIS Method

**DOI:** 10.3390/plants15060900

**Published:** 2026-03-13

**Authors:** Juntao Liu, Fang Xu, Xinyu Chen, Yingkai Wang, Ziping Deng, Qingsong Bai, Huanqin Liao, Baozhu Zhu, Weihua Zhang

**Affiliations:** Guangdong Provincial Key Laboratory of Silviculture, Protection and Utilization, Guangdong Academy of Forestry, Guangzhou 510520, China; ljt1120@bjfu.edu.cn (J.L.);

**Keywords:** environmental factors, entropy-weighted TOPSIS method, endangered plants, photosynthetic properties, *Michelia guangdongensis*

## Abstract

Photosynthesis is of pivotal significance to the growth, development, and survival of plants. The conservation of endangered plant species represents a significant challenge within the broader context of biodiversity conservation. Analysis of the photosynthetic, physiological, and ecological characteristics of endangered plants is of significant value in understanding their survival mechanisms under adverse conditions, and can also provide key scientific support for the development of effective conservation measures. This study selected one-year-old *Michelia guangdongensis* seedlings to measure diurnal photosynthetic variations across different asexual lines. Using entropy-weighted TOPSIS evaluation and statistical analysis, we aimed to investigate photosynthetic differences among asexual lines and their relationship with environmental factors, thereby revealing the physiological characteristics of photosynthesis in *M. guangdongensis* seedlings. The results showed that the daily net photosynthetic rate variation of asexual lines CG3 and 1 of *M. guangdongensis* had asymmetric double-peak curves. Moreover, strains 9073, 8898, 8812, and 5 showed single-peak curves. Leaf transpiration rate (Trmmol) and CO_2_ concentration (Ca) pathway analysis indicated these as the primary factors influencing the net photosynthetic rate of M. guangdongensis, with effective values of 1.17 and 0.9, respectively. TOPSIS entropy-weighted analysis indicated that the Ci values for CG3 and Leaf 1 exceeded 0.7, which indicated strong photosynthetic capacity. Additionally, the underside of the leaves exhibited superior coloration, thereby enhancing their ornamental value. This study provides a scientific basis and practical guidance for the breeding of asexual lines of *M. guangdongensis*.

## 1. Introduction

Photosynthesis is one of the most vital physiological processes for plant growth. It not only provides plants with essential energy and oxygen but also reflects their life habits [1,2]. Since photosynthesis is influenced by both intrinsic and environmental factors, investigating the relationship between plant photosynthesis and environmental factors not only provides fundamental theoretical support for transplanting and variety selection but also serves as an effective approach to elucidate the ecological adaptation mechanisms of different plants to their living environments [3,4]. Notably, the subtropical region has witnessed a continuous increase in the number of high-temperature days, accompanied by a significant upward trend in the frequency of intense solar radiation events. This characteristic change aligns closely with the annual growth rate of heatwave frequency and the trend of enhanced solar radiation observed in southern China [5,6]. This situation poses significant challenges to plant growth. It is directly linked to plants’ response strategies to environmental factors such as daylight and temperature, and has become a key window for revealing plant physiological rhythms, energy balance, and productivity potential [6,7]. However, excessive light and high temperatures can hinder plant growth and development [8]. Therefore, maintaining the efficiency and stability of photosynthesis under adverse conditions is particularly crucial for plant growth and development.

Light intensity and temperature are the core environmental factors regulating plant photosynthesis, and their mechanisms of action and ecological effects have long been a research focus in the field of plant physiology [7]. Research indicates that most plants exhibit significant heat adaptation characteristics within the optimal temperature range (0–32 °C) [9]. When temperatures exceed species-specific thresholds, plant photosynthetic systems will suffer multiple stresses, and high temperatures will cause abnormal stomatal behavior. For example, when rice plants undergo high-temperature stress, stomatal conductance decreases significantly, accompanied by an abnormal increase in intercellular carbon dioxide concentration [10]. It is noteworthy that the temperature effect exhibits marked species-specific differences. Temperate deciduous trees demonstrate significant photosynthetic advantages under moderate warming conditions (5–9 °C), as evidenced by substantially enhanced carbon assimilation, elevated net photosynthetic rates, and strengthened overall photosynthetic capacity. Therefore, the physiological changes in photosynthesis among different plants may be related to the degree of temperature variation [11]. In summary, various plants have evolved multilayered photoprotection strategies to cope with temperature fluctuations, such as chloroplast shading movements.

China possesses the world’s richest resources of *Magnoliaceae* plants, many of which are endangered species with scarce populations. For a long time, *Magnoliaceae* plants have been a popular subject in the fields of introduction and domestication [12,13,14,15,16]. The conservation of endangered plants has emerged as a critical focus in contemporary biological research, bearing profound significance for maintaining ecological equilibrium and safeguarding biodiversity. This study centered on *Michelia guangdongensis*, an endangered plant characterized by an extremely small population. A newly described species belonging to the genus Michelia (family Magnoliaceae), it is endemic to Guangdong Province, China. Distinguished by its delicate foliage and fragrant white blossoms, this species represents an exceptional candidate for ornamental landscaping applications. However, due to its minuscule population and fragile habitat, it faces an extremely high risk of extinction [17,18]. Photosynthesis, the core physiological process governing plant growth and development, has parameters that directly reflect a plant’s adaptive capacity to external environments and its physiological state. By thoroughly investigating the photosynthetic parameters of this endangered plant, we aimed to uncover its response strategies to specific environmental changes. This research will provide a scientific basis for developing effective conservation methods, holding significant practical importance for protecting this species’ population, promoting its recovery, and facilitating its expansion.

In this study, the chlorophyll content, diurnal variations in photosynthesis, and related parameters of leaves from different asexual lines of *M. guangdongensis* were measured under varying temperature and light conditions. The study investigated variations in photosynthetic efficiency among asexual lines under high-temperature and high-light conditions. Employing the entropy-weighted TOPSIS comprehensive analysis method, it provides a basis for selecting and breeding different asexual lines of *M. guangdongensis*, laying a theoretical foundation for breeding selection and cultivation promotion. Furthermore, this study establishes a theoretical foundation for the extensive application of *M. guangdongensis* in landscaping and ecological construction and provides crucial guidance for its future scientific cultivation and utilization.

## 2. Results

### 2.1. Daily Variation in Photosynthetic Parameters in Different Asexual M. guangdongensis Lines

**Figure 1:**
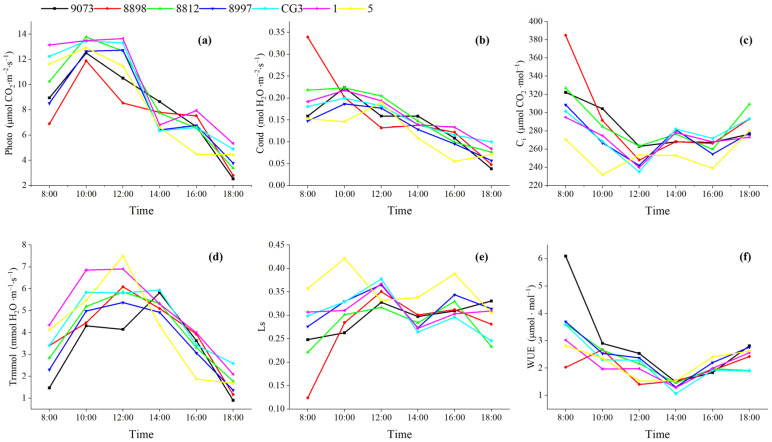
Daily variation curves of various photosynthetic parameters in leaves of different asexual *M. guangdongensis* lines. Note: (**a**) Net photosynthetic rate (photo); (**b**) stomatal conductance (Cond); (**c**) intercellular carbon dioxide concentration (Ci); (**d**) transpiration rate (Trmmol); (**e**) stomatal limiting value (Ls); (**f**) water use efficiency (WUE).

**Figure 2:**
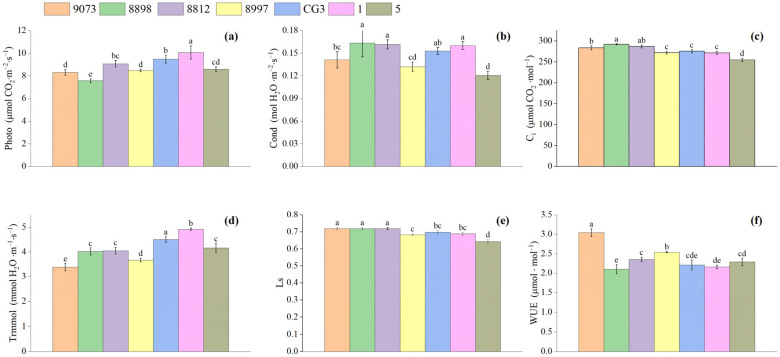
Daily average values of various photosynthetic parameters in leaves of different asexual *M. guangdongensis* lines. Note: (**a**) Net photosynthetic rate (photo); (**b**) stomatal conductance (Cond); (**c**) intercellular carbon dioxide concentration (Ci);(**d**) transpiration rate (Trmmol); (**e**) stomatal limiting value (Ls);(**f**) water use efficiency (WUE). Differences between different asexual lines are indicated by different lowercase letters.

The net photosynthetic rate serves as a key indicator for measuring the intensity of plant photosynthesis. In Figure 1a, the asexual *M. guangdongensis* lines 9073, 8898, 8812, and 5 exhibit a single-peak pattern of net photosynthesis rates. The results indicated that none of the plants exhibited a photosynthetic “midday break,” with their net photosynthetic rates rising steadily between 8:00 (local standard time; all subsequent times are local standard time) and 10:00, and peaking between 10:00 and 12:00 under light intensity. After 12:00, the net photosynthetic rate continued to decline due to environmental factors such as reduced light intensity and decreasing temperatures, reaching its lowest value at 18:00. The diurnal variation curves of the net photosynthetic rates of 8997, CG3, and 1 asexual lines exhibit an asymmetric double-peak pattern. The daily average net photosynthetic rates ranked in descending order was 1 > CG3 > 8812 > 5 > 8997 > 9073 > 8898 (Figure 2).

Stomata serve as vital pathways for carbon dioxide and water vapor to enter and exit plant leaves. The condition of a plant’s stomata directly impacts its growth and development. Figure 1b displays the diurnal variation curves for asexual lines 9073, 8812, 8997, CG3, and 1. These lines exhibit a single-peak pattern, with peaks occurring between 10:00 and 12:00. In contrast, asexual line 5 displayed an asymmetric double-peak pattern, with peaks distributed between 8:00 and 12:00. Asexual line 5 exhibited a single-peak pattern in its diurnal stomatal conductance curve, with the peak occurring between 10:00 and 12:00. Furthermore, line 5 displayed an asymmetric double-peak curve, with peaks occurring between 8:00 and 12:00. Figure 2 shows the daily average Gs values, ranked 8898 > 8812 > 1 > CG3 > 9073 > 8997 > 5.

The transpiration rate of leaves reflects a crop’s ability to regulate its own water loss under specific environmental conditions and is closely related to environmental and climatic factors. Generally, higher temperatures lead to stronger transpiration; however, when temperatures exceed a critical threshold, stomata close, causing transpiration to weaken. As shown in Figure 1d, the daily transpiration rate (Tr) curves for seven asexual lines exhibited distinct single-peak patterns, with peaks occurring between 12:00 and 14:00 and troughs at 18:00. In Figure 2, the daily average transpiration rates (Tr) were ranked 1 > CG3 > 5 > 8812 > 8898 > 8997 > 9073.

Ci is the external carbon dioxide concentration entering leaf cells. Figure 1c shows the diurnal variation curves of intercellular CO_2_concentration (Ci) for different clonal lines, which exhibited V-shaped and W-shaped patterns. These diurnal variation curves differed from other photosynthetic parameters like Photo, Gs, and Tr. The daily variation curves of Ci values for the asexual lines 9073, 8898, 8812, 8997, CG3, and 1 all exhibited a similar W-shaped pattern: first declining, then rising, followed by another decline, and finally rising again. These events occurred from 8:00 to 10:00 and 18:00 to 20:00. Finally, the diurnal variation curve of 5 exhibited a V shape, first declining and then rising (Figure 2). Besides the daily average intercellular carbon dioxide concentration (Ci) ranked 8898 > 8812 > 9073 > CG3 > 8997 > 1 > 5.

Figure 1e shows the daily variation trend of the stomatal closure threshold. The results closely resembled those of stomatal conductance (Gs). The Ci curves for asexual lines 9073, 8898, 8812, 8997, CG3, and 1 exhibited a W-shaped characteristic: first decreased, then increased, followed by another decrease and increase. Figure 2 shows the daily average stomatal limitation values, ranked 8898 > 8812 > 9073 > CG3 > 1 > 8997 > 5.

Except for the asexual line 8898, water use efficiency across different asexual lines typically exhibited a V-shaped diurnal variation pattern, peaking in the early morning, afternoon, and evening (Figure 1f). Significant differences existed in the trends of water use efficiency during the 8:00–10:00 period. The six asexual lines exhibited a declining trend in water use efficiency, while other asexual lines showed an increasing trend. This could be due to the higher humidity of southern regions and moisture content in the early morning, coupled with more pronounced fluctuations in light intensity and vapor pressure. Additionally, discrepancies between the microenvironment during photosynthetic measurements and prevailing environmental conditions contributed to variations in water used efficiency. Between 10:00 and 12:00, the variation in these differences gradually decreased. However, after 14:00, the water use efficiency increased slightly. The daily average water use efficiency decreased as follows: 9073 > 8997 > 8812 > 5 > CG3 > 1 > 8898.

Figure 2 shows the photosynthetic values of CG3 and 1. The results were higher compared to the 8812, 5, and 8997 lines. In addition, both photosynthetic values were higher than those of 9073 and 8898 lines. According to an analysis of variance, significant differences (*p* < 0.05) existed among the seven asexual lines in their photosynthetic parameter characteristics within the species.

### 2.2. Influence of Environmental Factors on Photosynthetic Parameters of M. guangdongensis Asexual Lines

**Figure 3:**
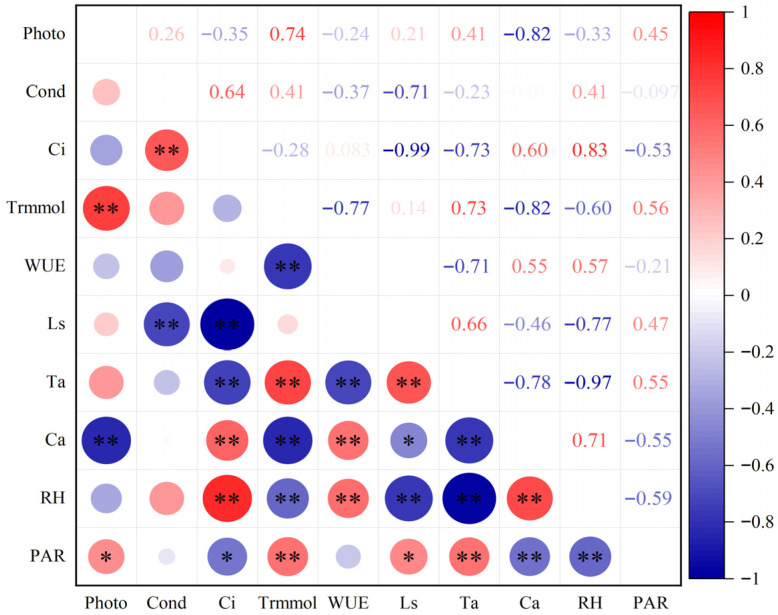
Correlation of different asexual *M. guangdongensis* lines with various indicators. * *p* < 0.05; ** *p* < 0.01. Note: photosynthetically active radiation (PAR); air temperature (Ta); relative humidity (RH); CO_2_ concentration (Ca).

Relationships between daily average photosynthetic parameters of leaves were investigated and their physiological and ecological factors correlated with Pearson correlation analysis (see Figure 3). The results indicated that photosynthetic rate (Photo) showed significant positive correlations with transpiration rate (Trmmol) and photosynthetically active radiation (PAR). Furthermore, it exhibited significant negative correlations with CO_2_ concentration (Ca) and intercellular carbon dioxide concentration (Ci). Leaf stomatal conductance (Cond) demonstrated a significant positive correlation with Ci and a negative correlation with water use efficiency (WUE). Canopy transpiration showed significant positive correlations with temperature, light intensity, and relative humidity, and significant negative correlations with precipitation, temperature, and photosynthetically active radiation. Water use efficiency exhibited significant positive correlations with temperature and relative humidity and highly significant negative correlations with canopy transpiration and temperature. Photosynthetic parameters (Ci, Trmmol, WUE, Ls) exhibited distinct significant positive or negative correlations with environmental ecological factors (Ta, Ca, RH, PAR): Ta showed significant positive correlations with PAR, Ls, and Trmmol, and significant negative correlations with RH, Ci, WUE, Ca, and Ls. The results indicated that photosynthetic parameters of different asexual *M. guangdongensis* lines exhibited extremely close correlations with physiological and ecological factors. Simultaneously, the study revealed that photosynthetic traits in plants interact with and are constrained by one another.

### 2.3. PCA of Photosynthetic Parameters and Environmental Factors in M. guangdongensis Leaves

**Figure 4:**
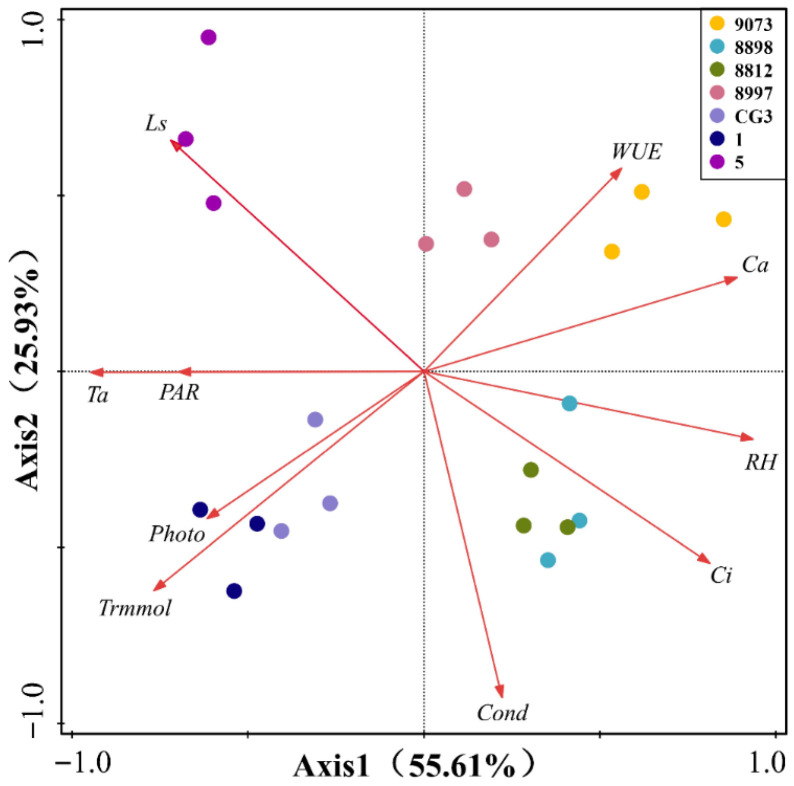
Principal component analysis of photosynthetic characteristics of leaves in different asexual *M. guangdongensis* lines.

Principal component analysis of photosynthetic parameters and environmental factors explained 81.54% of the variance along the first two principal component axes (see Figure 4). It retained most of the information from the original data and better reflected the utilization and distribution of resources during plant growth. The first principal component was the photosynthetic parameters of plant leaves, which explained 55.61% of the variance. These parameters correlated with Photo, Ci, Trmmol, Ta, Ca, RH, and PAR. Also Photo was positively correlated with Trmmol, PAR, and Ta and negatively correlated with WUE, Ca, RH, and Ci. From right to left along the first principal component, there is a gradient of variation, since WUE, Ca, RH, and Ci gradually decreased. Besides Photo, Trmmol, PAR, and Ta gradually increased. The second PCA axis explained 25.93% of the variance. It was correlated with Cond and WUE. The species-trait ordination plot revealed convergent divergence in photosynthetic parameters and environmental factor indicators (statistical analysis).

### 2.4. Pathway Analysis

Pathway analysis of the direct and indirect effects of external environmental factors on the net photosynthetic rate of *M. guangdongensis* leaves indicated that Trmmol exhibited the highest direct positive effect on the net photosynthetic rate of leaves (1.17), followed by Ca (0.9) and RH (0.26). Indirect pathway coefficients revealed that external environmental factors contributed to the net photosynthetic rate of *M. guangdongensis* leaves to some extent, as well as other factors (Table 1).

### 2.5. Multi-Objective Decision-Making and Evaluation Based on Entropy-Weighted TOPSIS Approach

An entropy-weighted TOPSIS model was employed to comprehensively evaluate the photosynthetic trait indices of leaf samples from different clonal lines of the endangered plant *M. guangdongensis*. The results are show in Table 2. The TOPSIS of *M. guangdongensis* clonal lines data ranked 1 > CG3 > 8812 > 8898 > 9073 > 5 > 8897. CG3 and 1 demonstrated outstanding performance in the comprehensive evaluation of leaf photosynthetic capacity, with both exhibiting stomatal conductance values exceeded 0.7. Observations revealed these cultivars possess exceptional ornamental characteristics, primarily manifested in more visually appealing underside leaf coloration, larger leaves, denser pubescence, and deeper, richer hues.

### 2.6. Linear Fitting of Photosynthetic Indices to Chlorophyll Content

**Figure 5:**
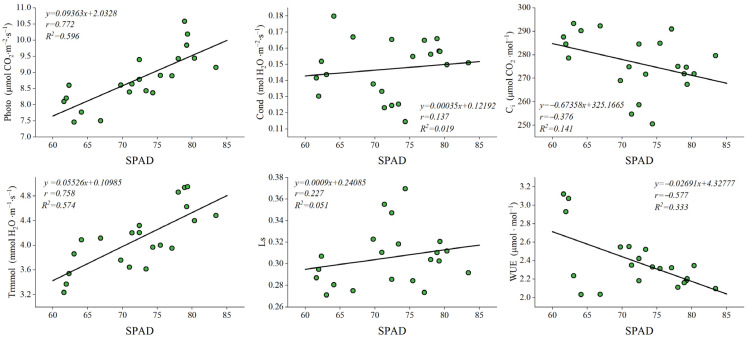
Linear regression fit of leaf photosynthetic characteristics to chlorophyll content.

Chlorophyll content exhibited a significant positive correlation with net photosynthetic rate and transpiration rate, in addition to a significant negative correlation with water use efficiency (Figure 5). It also showed a non-significant positive correlation with stomatal conductance and stomatal limitation and a non-significant negative correlation with intercellular CO_2_ concentration. This indicated that leaf chlorophyll content is related to photosynthetic parameters, and suggested indirectly that chlorophyll levels determine a tree’s photosynthetic capacity and growth status. Therefore, chlorophyll content can serve as a key technical indicator for the selection and breeding of ornamental early-leaf varieties of *M. guangdongensis*.

## 3. Discussion

### 3.1. Effect on Leaf Color on M. guangdongensis Photosynthesis Variation

The diurnal variation in plant photosynthetic indices reflects the patterns of photosynthetic activity in response to changing environmental conditions and the adaptability of plants to environmental shifts [19]. Overall, the diurnal variation in plant photosynthesis is typically categorized into two curve types: single peak and double peak [20]. This study revealed that different asexual lines of *M. guangdongensis* exhibited fundamentally similar diurnal patterns in net photosynthetic rate, transpiration rate, and stomatal conductance, consistent with the findings of Liu Xi et al. [21]. Net photosynthetic rate diurnal variation curves of 9073, 8898, 8812, and 5 all exhibited single-peak patterns, showing no distinct midday depression of photosynthesis, with 9073 and 8898 having lower photosynthetic pigment content, lighter leaf coloration, and reduced chlorophyll content. Net photosynthetic rate diurnal variation curves of asexual lines 8997, CG3, and 1 exhibited asymmetric double-peaked patterns, demonstrating pronounced midday depression of photosynthesis. Asexual lines CG3 and 1 displayed dark leaf coloration with high chlorophyll content, resulting in significantly elevated net photosynthetic rates.

Under normal conditions, leaves maintain a strong photosynthetic carbon assimilation capacity, which allows them to recover photosynthetic function more rapidly after abiotic stress events such as midday heat exposure. This resilience may be attributed to the variety’s ability to dissipate excess absorbed light energy as heat, thereby indirectly reducing stress-induced damage to the leaves. This enables rapid recovery and sustained high levels of photosynthesis. A regulatory mechanism within plants, the “midday depression of photosynthesis” plays a positive role in plant survival and growth under adverse conditions [22]. Research indicates that the midday depression of photosynthesis is primarily caused by both stomatal and non-stomatal limiting factors. In the theory proposed by Farquhar and Scurlock, this phenomenon is mainly attributed to the combined effects of stomatal and non-stomatal limiting factors [23,24]. When the intercellular carbon dioxide concentration decreases while the stomatal limiting value increases, it can be inferred that the decline in net photosynthetic rate during midday is primarily attributed to stomatal factors. Conversely, if the decline in leaf photosynthetic rate is accompanied by an increase in intercellular CO_2_ concentration, non-stomatal factors may be the primary limiting factor for photosynthesis. In this study, we found that during the midday depression of photosynthesis, net photosynthetic rates significantly decreased in the leaves of CG3 and 1, while stomatal conductance slightly declined and intercellular CO_2_ concentration slightly increased. These trends in photosynthetic parameters indicate that the photosynthetic “midday depression of photosynthesis” phenomenon in asexual line CG3 and 1 is primarily caused by non-stomatal limiting factors. The formation of C3 and C4 plants stems from differences in their photosynthetic carbon assimilation capabilities. Photosynthesis is a highly conserved physiological process in plant evolution, enabling plants to adapt to their environment through morphological and pigment changes [25]. Research indicates that the net photosynthetic rate of asexual *M. guangdongensis* lines peaks between 10:00 and 12:00, consistent with findings from studies on other tree species, such as *Populus tomentosa* Carrière [26], *Carya cathayensis* Sarg [27], *Amorpha fruticosa* L. [28], and *Michelia maudiae* Dunn [29]. The net photosynthetic rates of asexual *M. guangdongensis* lines 9073 and 8898 were lower than those of other asexual lines. This may be attributed to reduced net photosynthetic rates and elevated intercellular CO_2_ concentrations caused by low temperatures and weak light in the early morning. As light intensity and temperature increase, the combined effects of stomatal closure and limited carbon dioxide supply inhibit the dark reaction, leading to a decrease in net photosynthetic rate. Research indicates that under conditions of intense light and high temperatures, plants regulate stomata to limit photosynthesis, thereby reducing water loss through transpiration [30,31]. After 14:00, light intensity and temperature decrease, leading to reduced net photosynthetic rate and stomatal conductance, while intercellular carbon dioxide concentration rises [32]. The asexual lines CG3 and 1 exhibit higher average photosynthetic rates, transpiration rates, and stomatal conductance along with lower water use efficiency, indicating strong adaptability to high temperatures and intense light conditions, as well as substantial photosynthetic potential. When promoting its cultivation, appropriate resources should be selected based on local water and temperature conditions.

### 3.2. Effect on Environmental Factors Between Photosynthesis Parameters

The net photosynthetic rate of plants is influenced by multiple factors, including environmental and physiological factors [33,34,35,36]. The results of this study indicate that the net photosynthetic rate of *M. guangdongensis* exhibits a significant positive correlation with Trmmol and PAR, a significant negative correlation with Ca, and a negative correlation with Ci. Stomata play a regulatory role in tree water loss and carbon dioxide gas exchange, with Trmmol being the primary factor influencing net photosynthetic rate. Stomatal conductance showed a significant positive correlation with Trmmol and negative correlations with leaf stomatal resistance and water use efficiency. This indicates that when stomatal conductance decreases, resistance to water loss from leaves increases, transpiratory activity weakens, and transpiration rates decline, thereby adapting to arid environments [28,29]. Research indicates that the ultimate source of energy for plant photosynthesis is photosynthetically active radiation, and insufficient light energy limits photosynthetic activity [37]. According to stepwise regression analysis, the primary photosynthetic parameters and physiological-ecological factors influencing the net photosynthetic rate of *M. guangdongensis* asexual lines are stomatal conductance, transport rate, leaf area, water use efficiency, leaf temperature, and photosynthetically active radiation. Research indicates that as time progresses and both photosynthetically active radiation and temperature in the external environment increase, relative air humidity gradually decreases. This leads to elevated plant stomatal conductance and transpiration rates, increased CO_2_ assimilation rates, and reduced intercellular CO_2_ concentrations. Consequently, leaf photosynthetic enzyme activity continues to increase, resulting in a corresponding increase in net photosynthetic rate [38]. The pathway analysis showed that Trmmol, Ls, and PAR were the main factors affecting the net photosynthetic rate, which was consistent with the results of *V. purpurea*, studied by Yuan Shan [39]. Changes in external environmental factors significantly impact plant photosynthesis, while the efficiency of photosynthetic energy utilization is influenced by multiple factors, including leaf morphological structure (leathery leaves), chlorophyll content, and transpiration rate intensity [40]. Chlorophyll content is the key pigment involved in the conversion, transfer, and absorption of light energy during photosynthesis. Consequently, the photosynthetic rate of leaves is closely related to chlorophyll content. The results of this study indicate that chlorophyll content exhibits a significant positive correlation with net photosynthetic rate and transpiration rate and a significant negative correlation with water use efficiency. Therefore, chlorophyll content levels play a decisive role in the photosynthetic capacity and growth of *M. guangdongensis*. Further research indicates that between 12:00 and 14:00, the water use efficiency of CG3 and1 varieties decreased more significantly. This may be related to photosynthetically active radiation (PAR) among environmental factors, as changes in PAR affect air temperature and humidity, which in turn influence transpiration rates. When photosynthetically active radiation and air temperature increase, transpiration intensifies, leading to heightened water consumption in leaves and consequently reducing water use efficiency. However, water use efficiency improved between 14:00 and 16:00, indicating that photosynthetically active radiation and air temperature decreased, plant root water uptake capacity increased, and the plants’ adaptability to external environmental changes also improved. It can be seen that the plants’ trade-off strategy between water use efficiency and photosynthetic efficiency reflects an optimized model of environmental adaptation and resource utilization efficiency. Based on the characteristics of the wild habitat of *M. guangdongensis* in Guangdong, we propose a possible explanation: although the province’s overall high atmospheric humidity and ample sunlight are favorable for most plant growth, excessively high humidity and solar radiation intensity may cause stomatal closure. This in turn impairs photosynthesis and gas exchange, reducing water use efficiency and leading to overall plant dehydration that threatens survival, particularly during summer months. This phenomenon may be one reason that *M. guangdongensis* has become an endangered plant species.

This study measured and analyzed diurnal variations in photosynthetic parameters of endangered plants. When light conditions change, core parameters such as photosynthetic rate and stomatal conductance undergo rapid and orderly adjustments [7]. This represents an adaptive strategy shaped by long-term natural selection, enabling plants to maximize light energy utilization while avoiding damage to their photosynthetic apparatus. By precisely analyzing changes in these physiological parameters, researchers can define the range of light conditions to which plants are optimally adapted, thereby providing a scientific basis for developing effective light management protocols. Furthermore, if drought is found to inhibit photosynthesis, humidity can be improved through precision irrigation. Given the close correlation between photosynthetic parameters and nutritional status, changes in these parameters can also indicate nutrient requirements. This allows for rational fertilization, providing plants with balanced nutrients to ensure their normal growth and development.

### 3.3. Recommendations for Variety Selection and Introduction Measures

Commonly used methods in integrated crop and plant evaluation primarily include membership function analysis, principal component analysis, the TOPSIS method, analytic hierarchy process, and gray correlation analysis [41,42,43]. This study employed the entropy-weighted TOPSIS method to conduct a comprehensive evaluation of the photosynthetic characteristics of different asexual *M. guangdongensis* lines. This method was selected for its advantages in multi-attribute decision-making, particularly for addressing problems involving multiple evaluation criteria. Compared with other comprehensive evaluation methods, the entropy-weighted TOPSIS approach combines the objectivity of the entropy-weighted method with the multi-objective optimization capability of the TOPSIS method. It partially overcomes the issue of the subjective weighting inherent in traditional TOPSIS and compensates for the shortcomings of principal component analysis or factor analysis, which cannot perform indicator standardization. Relevant studies indicate that the entropy-weighted TOPSIS method has been applied to the comprehensive evaluation of various plants and cash crops. For instance, research by Jiazhen Hu et al. [44] and Zijian He et al. [45] confirmed the method’s effectiveness in optimizing rice (*Oryza sativa* L.) fertilization strategies [44] and apple (*Malus pumila* Mill.) fertilization schemes. Similarly, Juin Yau Lim et al. [46] demonstrated the application of this method in evaluating the multi-objective life cycle optimization of formulated fertilizers for oil palm. This research further expands the scope of this methodology, applying it for the first time to assess the photosynthetic characteristics of asexual *M. guangdongensis* lines. The entropy-weighted TOPSIS method was employed to comprehensively evaluate the photosynthetic parameters of different asexual *M. guangdongensis* lines. Through this approach, CG3 and CG1 were identified as superior lines, both exhibiting Ci values above 0.7. These lines demonstrated superior leaf-back coloration with deeper hues and enhanced ornamental value.

Referencing the concept of the leaf economic spectrum proposed by Wright et al. [47], this model quantifies a continuum of plant resource trade-off strategies by integrating interrelated and synergistic traits. Based on principal component analysis results, CG3 and 1 exhibit darker leaf coloration and occupy the same quadrant, characterized by high photosynthetic and transpiration rates, collectively displaying “fast-return” species traits. The remaining species exhibit high water use efficiency coupled with low photosynthetic efficiency, classifying them as “slow investment-return” types. It can be observed that different species of *M. guangdongensis* exhibit variations in photosynthetic resource allocation and investment strategies. Research by Matthew et al. [48] indicates that leaf economic traits reveal correlations among leaf characteristics and the mechanisms by which environmental factors influence them, reflecting the trade-offs between resource acquisition and conservation strategies in plant species. However, the study failed to adequately explore the influence of intraspecific variation on trait correlations within tree species. The experimental materials selected in this study reflected intraspecific variation through leaf color characteristics, and the relationship between different leaf colors and environmental factors in *M. guangdongensis* was analyzed. The path analysis results indicate that Trmmol, Ls, and PAR are the primary factors influencing the net photosynthetic rate of *M. guangdongensis*. This suggests that external environmental factors play a crucial role in shaping its photosynthetic performance and adaptive strategies, highlighting the complexity of species variability in responding to dynamic environmental conditions.

As an endangered species in China, wild *M. guangdongensis*’s distribution range and population size are minimal, as is systematic research on it. Studies on the photosynthetic characteristics of *M. guangdongensis* are particularly rare in China.

This study conducted a comprehensive analysis of the photosynthetic characteristics of seven asexual lines of *M. guangdongensis*. The results indicated that these asexual lines exhibit strong adaptability to higher temperatures and light intensities, with significantly enhanced photosynthetic capacity. To increase the photosynthetic rate of *M. guangdongensis* and mitigate the impact of midday photosynthetic depression photosynthesis, attention should be paid to light conditions across different seasons. For mature trees, appropriate pruning is required to enhance sunlight exposure. During periods of high temperatures, intensify water and fertilizer management while applying foliar sprays of appropriate amounts of transpiration inhibitors to reduce plant transpiration, improve water use efficiency and photosynthetic efficiency, and promote rapid growth. As light intensity and temperature gradually decrease, the focus should be on thinning branches and foliage to maintain ventilation. This ensures plants receive sufficient light and further improves photosynthetic efficiency, maximizing their ability to utilize available light energy.

Building on this foundation, we will conduct more systematic research and provide comprehensive guidance on the standardized cultivation of seven species of *M. guangdongensis*.

### 3.4. Limitation and Outlook

This study adopted the entropy-weighting method to determine weight coefficients, thereby minimizing the impact of subjective judgment on evaluation results. The merit of this approach is that it assigns weights according to the information inherent to the data, which enables more objective and reliable assessment outcomes. Our research findings indicate that the entropy-weighted TOPSIS method can effectively distinguish the photosynthetic characteristics of different asexual *M. guangdongensis* lines, providing a scientific basis for the selection and breeding of superior asexual lines. However, we also recognize that the entropy-weighted TOPSIS method is not without limitations. First, this method relies on the quality and completeness of the data—missing or noisy data may compromise the accuracy of the evaluation results. Second, while the entropy-weighting method reduces subjectivity, it may fail to adequately account for interactions among evaluation indicators and the influence of expert knowledge. This study demonstrates that the entropy-weighted TOPSIS method serves as an effective tool for evaluating the photosynthetic characteristics of asexual *M. guangdongensis* lines, providing important reference for future breeding efforts. However, subsequent research should consider the limitations of this method in practical applications and explore potential improvement strategies.

## 4. Materials and Methods

### 4.1. Overview of the Trial Site

**Figure 6:**
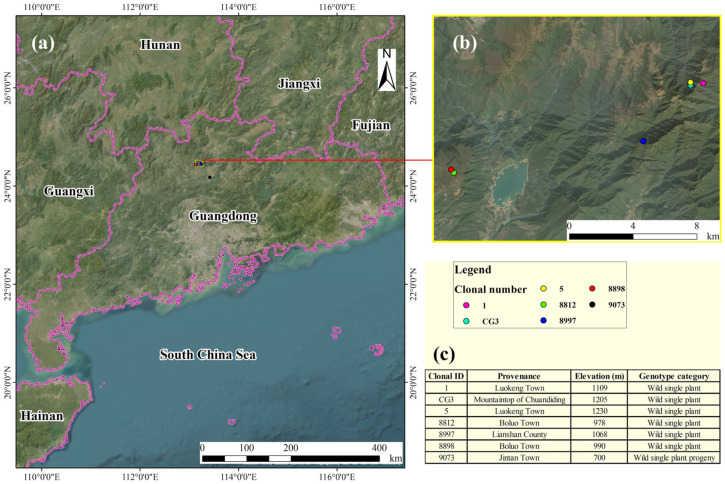
Geographic location of different sources.Note: (**a**,**b**) indicate the planting locations of individual wild *M. guangdongensis* trees in Guangdong, while (**c**) indicates the specific planting locations of different *M. guangdongensis* lines.

**Figure 7:**
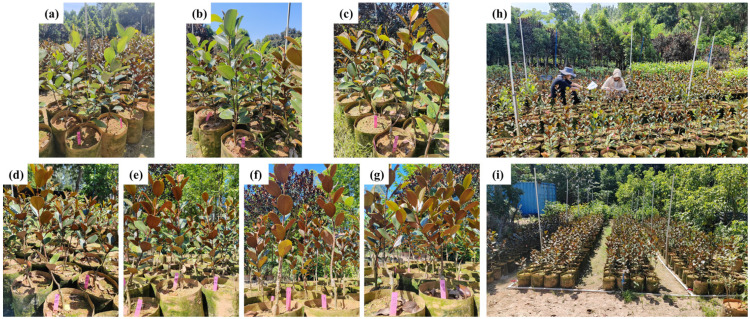
Photographs of different asexual *M. guangdongensis* lines: (**a**) 9073; (**b**) 8898; (**c**) 8812; (**d**) 8997; (**e**) CG3; (**f**) 1; (**g**) 5. (**h**) Photosynthesis was measured using an Li-6400XT. (**i**) Nursery environments in which *M. guangdongensis* was cultivated.

The experimental site was the Guangdong Academy of Forestry Sciences Nursery (113°22′6″ E, 23°11′59″ N). This region features a subtropical monsoon climate characterized by warm temperatures, abundant rainfall, and distinct seasons. Summers are hot and rainy, while winters are mild and dry. The annual average temperature is 23 °C, with an annual average rainfall of 1683 mm. In January 2024, superior scions of Pholiota orientalis (*Michelia macclurei* Dandy) were selected from the Guangdong Shimen Tai National Nature Reserve. Two-year-old *Michelia macclurei* Dandy seedlings were chosen as rootstocks for grafting propagation. By May, the grafted seedlings had grown to over 10 cm in height. They were planted in nutrient bags measuring 25 cm × 25 cm with a substrate mixture of humus soil–red soil = 1:3 (by volume). A total of 38 asexual lines were grafted in this trial, from which 7 exhibiting distinct characteristics were selected and numbered as follows: 9073, 8898, 8812, 8997, CG3, 1, and 5. Each asexual line comprised three replicates, with four plants per replicate. Routine maintenance practices such as regular weeding, watering, and balanced fertilization were identical to conventional cultivation methods, resulting in vigorous seedling growth. For the experiment, one-year-old grafted seedlings from different asexual lines with comparable growth vigor in the middle to upper sections of each pot were selected (Figure 6 and Figure 7).

Grafting process and method: Make a downward diagonal cut on the back of the scion bud, cutting just deep enough to reach the woody tissue. Slice the base of the bud into a smooth, flat surface. Then, make a short diagonal cut on the back of the tapered surface. At this point, the cut end of the scion should form a wedge shape. For rootstock preparation, select an appropriate height to cut off the top of the rootstock. On the side with thicker bark and a smooth, straight trunk, make a straight downward cut with a grafting knife. The size of the cut should be equal to or slightly larger than the scion’s cut surface. Carefully insert the scion’s cut surface into the rootstock’s grafting slot, ensuring the longer sides of the tapered surface align perfectly with the rootstock’s cambium layer and fit snugly. Finally, secure the graft tightly with plastic wrap.

In this study, we used meteorological data from Guangzhou. Following consecutive weeks of temperatures exceeding 40 °C in summer field nurseries accompanied by sustained sunny and hot weather (with daytime highs reaching 43 °C), vigorously growing plants were chosen for testing in open-air sites with no surrounding shade. Measurements were conducted from 8:00 to 18:00, with data recorded at two-hour intervals.

### 4.2. Methods for Determining Photosynthetic Characteristics and Leaf Color

In early September 2024, under continuous sunny conditions, daily photosynthetic changes in the leaves of *M. guangdongensis* were measured using a Li-6400XT portable photosynthesis meter (LI-COR, Lincoln, NE, USA) between 8:00 and 18:00. Leaves with consistent orientation, intact structure, and free of pests or diseases were selected for measurements taken every two hours. Leaf SPAD values were measured using a portable chlorophyll meter (SPAD-502, Konica Minolta, Japan), with measurement periods and locations synchronized with leaf photosynthetic parameter assessments.

The saturated light intensity was set to 1000 μmol/(m^2^·s), and the air flow rate was 500 μmol/s. The physiological. factors included net photosynthetic rate (μmol CO_2_ m^−2^s^−1^), stomatal conductance (mol H_2_O m^−2^s−^1^), intercellular carbon dioxide concentration (Ci, μmol CO_2_ mol^−1^), and transpiration rate (Tr, mmol H_2_O m^−2^s^−1^). The net photosynthetic rate/transpiration rate was used to calculate water use efficiency (WUE, μmol/mmol) and stomatal limiting value (Ls = 1 − (Ci/Ca)). The environmental factors included photosynthetically active radiation (PAR), air temperature (Ta), atmospheric relative humidity (RH), atmospheric CO_2_ concentration (Ca), and other indices [49,50].

**Figure 8:**
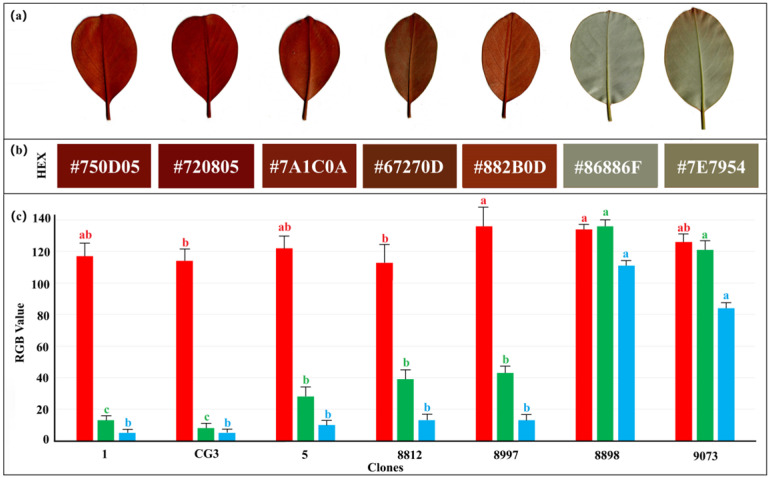
Leaf color variation in different asexual *M. guangdongensis* lines. Note: (**a**) Leaf Colour Variation in Different Clonal Lines of Michelia guangdongensis (**b**) HEX code to the leaf colour (**c**) RGB value changes of leaves, each bar represents the corresponding color component, and letters indicate significant differences from multiple comparisons.

First, collect leaves from the seedling room for imaging. Scan the leaves using a HP DeskJet 2622 Multifunction scanner (HP, Beijing, China) with 1920 × 1080 pixels and save them in JPEG format. Subsequently, color parameters of the leaf images were extracted. Using ImageJ2 (National Institutes of Health R&D) software, the leaf images were segmented to remove the background and retain the main leaf structure. This study employed MATLAB 2021 (R2021a Version V9.10) to design an image processing method that automatically extracted leaf color features based on a set color-threshold range, thereby filtering pixels meeting the criteria from the images. The red, green, and blue channels of the leaf images were captured in a software histogram (Figure 8).

### 4.3. Entropy-Weighted TOPSIS Method for Comprehensive Evaluation

The technique for ordering of preference by similarity to ideal solution (TOPSIS) method determines a feasible solution set by defining the optimal and worst solutions to a problem and selects the optimal solution furthest from the optimal solution [51,52]. The analysis process is as follows.

(1) The evaluation index matrix for growth traits of asexual lines of different forest tree species is established as follows.

$$X=\left[\begin{array}{ccccc} X_{11} & \ldots & X_{1j} & \ldots & X_{1m}\\ \ldots & & \ldots & & \ldots \\ X_{i1} & \ldots & X_{ij} & \ldots & X_{im}\\ \ldots & & \ldots & & \ldots \\ X_{n1} & \ldots & X_{nj} & \ldots & X_{nm} \end{array}\right]$$

Here, X_ij_ denotes the jth evaluation index of the ith asexual line in the original data, where n equals 7 (number of asexual lines) and m equals 4 (number of photosynthetic parameter indices).

(2) Evaluation indicators are standardized to harmonize the types and dimensions of each indicator, with the following formulae.

For positive indicators, use the following formula:

$$X_{ij}^{\prime }=\frac{X_{ij}-max\left(X_{1j},X_{2j},\ldots ,X_{ij}\right)}{\max\left(X_{1j},X_{2j},\ldots ,X_{ij}\right)-min(X_{1j},X_{2j},\ldots ,X_{ij})}+1$$

For negative indicators, the following formula is used:

$$X_{ij}^{\prime }=\frac{\max\left(X_{ij},X_{2j},\ldots ,X_{ij}\right)-X_{ij}}{\max\left(X_{ij},X_{ij},\ldots ,X_{ij}\right)-min(X_{1j},X_{2j},\ldots ,X_{ij})}+1$$

where X_ij_ is the jth indicator value of the ith asexual line (i=1,…,n;j=1,…,m) and X’_ij_ is standardized X_ij_.

(3) The proportion of the jth indicator represented by the ith asexual line (P_ij_) is calculated as follows:

$$P_{ij}=\frac{X_{ij}^{\prime }}{\sum_{i=1}^{n}X_{ij}}$$

(4) The entropy value e_j_ of the jth indicator is calculated as follows:

$$e_{j}=-\frac{\sum_{i=1}^{n}P_{ij}\ln\left(P_{ij}\right)}{\ln n}$$

(5) The coefficient of variation g_j_ for the jth indicator is calculated as follows:

$$g_{i}=1-e_{j}$$

(6) The weight of the jth indicator W_j_ is calculated as follows:

$$W_{j}=\frac{g_{i}}{\sum_{i=1}^{m}g_{i}}$$

(7) A weighted normalized decision matrix (R) is formed from the normalized decision matrix X = (X’_ij_) 7 × 4 and the weight vector W = (w_1_, w_2_, w_3_, …, w_7_):

$$R=(R_{ij}{)}_{m\times n}=(W_{j}X_{ij}{)}_{m\times n}$$

(8) Determine the optimal solution $Z_{ij}^{+}$ and the worst solution $Z_{ij}^{-}$ to form the optimal vector Z^+^ and the worst vector Z^−^, respectively, as follows:

$$Z_{ij}^{+}=(maxR_{i1}^{+},maxR_{i2}^{+},maxR_{i3}^{+},maxR_{i4}^{+})$$

$$Z_{ij}^{-}=(minR_{i1}^{-},minR_{i2}^{-},minR_{i3}^{-},minR_{i4}^{-})$$

(9) Determine the Euclidean spatial distances D^+^ and D^−^ between the optimal and worst solutions of the seven asexual lines:

$$D_{j}^{+}=\sqrt{\sum_{j=1}^{m}{\left[w_{j}\times (r_{ij}-Z_{ij}^{+})\right]}^{2}}$$

$$D_{j}^{-}=\sqrt{\sum_{j=1}^{m}{\left[w_{j}\times (r_{ij}-Z_{ij}^{-})\right]}^{2}}$$

(10) Calculate the performance evaluation value Ci for each asexual line, i.e., calculate the closeness of the evaluation object to the optimal solution as follows:

$$C_{i}=\frac{D_{i}^{-}}{D_{i}^{+}+D_{i}^{-}}$$

### 4.4. Statistical Processing

Statistical analysis and data visualization were implemented in R 4.4.3 Graphical representation was achieved using the ggplot2 package, primarily encompassing the following methods.

(1) Relationships between photosynthetic parameters and environmental factors were examined using principal component analysis (PCA) and Pearson correlation coefficients (calculated with the FactoMine R 4.4.3 software package) [53].
(2) Regression equations linking net photosynthetic rates to environmental factors were derived using stepwise linear regression analysis.
(3) Path analysis assessed both direct and indirect effects of physiological and ecological factors on net photosynthetic rates. Direct effects were obtained from direct path coefficients, while indirect effects were calculated using the equation of path coefficient × correlation coefficient [54,55].

## 5. Conclusions

This research will provide a scientific basis for developing effective conservation methods, holding significant practical importance for protecting this species’ population, promoting its recovery, and facilitating its expansion. In this study, the chlorophyll content, diurnal variations in photosynthesis, and related parameters of leaves from different asexual lines of *M. guangdongensis* were measured under varying temperature and light conditions. The study investigated variations in photosynthetic efficiency among asexual lines under high-temperature and high-light conditions. Employing the entropy-weighted TOPSIS comprehensive analysis method, it provides a basis for selecting and breeding different asexual lines of *M. guangdongensis* in landscaping and ecological construction, and provides crucial guidance for its future scientific cultivation and utilization. The following conclusions were drawn.

(1) Diurnal variation curves of net photosynthetic rate differ among asexual lines of *M. guangdongensis*. The curves for CG3 and 1 exhibit asymmetric “biphasic” patterns, whereas those for 8812 and 5 display “monophasic” curves.
(2) Pathway analysis indicates that the leaf transpiration rate (Trmmol) and CO_2_ concentration (Ca) are the primary factors influencing the net photosynthetic rate of *M. guangdongensis*.
(3) Photosynthetic midday depression of photosynthesis in leaves of different *M. guangdongensis* asexual lines (CG3, 1) is primarily driven by non-stomatal limiting factors.
(4) Comprehensive evaluation of these asexual *M. guangdongensis* lines (CG3, 1) reveals superior abaxial leaf coloration with deeper hues, indicating higher economic value and broader suitability for cultivation. Future research may consider integrating more advanced machine learning algorithms with comprehensive evaluation methods based on trait characteristics, such as leaf traits, nutritional components, growth status, and soil factors. Establishing correlations between these traits and economic value and ecological functions will provide a reliable assessment framework for predicting and enhancing the sustainable utilization and management of endangered plant resources.

## Figures and Tables

**Table 1:** Direct and indirect path coefficients between photosynthetic rates and their primary influencing factors in *M. guangdongensis*.

Parameter	Cond	Ci	Trmmol	WUE	Ls	Ta	Ca	RH	PAR
Cond	**0.05**	−0.92	0.68	0	0.44	0.07	0.24	0.1	0.04
Ci	0.02	**−2.3**	−0.36	−0.01	1.13	0.55	0.62	0.18	−0.03
Trmmol	0.03	0.7	**1.17**	0.03	−0.19	−0.57	−0.43	−0.11	0.06
WUE	0	−0.53	−0.62	**−0.05**	−0.05	0.58	0.63	0.19	−0.01
Ls	−0.02	2.16	0.19	0	**−1.21**	−0.39	−0.35	−0.13	0.03
Ta	0	1.64	0.85	0.04	−0.61	**−0.78**	−0.74	−0.23	0.03
Ca	0.01	−1.58	−0.56	−0.04	0.47	0.64	**0.9**	0.21	−0.01
RH	0.02	−1.64	−0.52	−0.04	0.6	0.69	0.74	**0.26**	−0.01
PAR	0.02	0.69	0.74	0	−0.39	−0.29	−0.13	−0.02	**0.09**

Bold numbers represent direct effect values.

**Table 2:** Comprehensive evaluation analysis of different *M. guangdongensis* asexual lines.

Serial Number	Euclidean Space Distance, D^+^	Euclidean Space Distance, D^−^	Closeness, Ci	Ranking
9073	0.36	0.21	0.37	5
8898	0.31	0.34	0.52	4
8812	0.19	0.35	0.65	3
8997	0.35	0.16	0.31	7
CG3	0.15	0.36	0.71	2
1	0.12	0.45	0.79	1
5	0.38	0.17	0.32	6

## Data Availability

The original contributions presented in the study are included in the article. Further inquiries can be directed to the corresponding authors.
